# Supplementary material for: Kaempferol Interferes with Varicella-Zoster Virus Replication in Human Foreskin Fibroblasts
Source: Pharmaceuticals (Basel). 2022 Dec 19;15(12):1582. doi: 10.3390/ph15121582 (PMC9788135; doi:10.3390/ph15121582)
Supplement: Supplementary file 1 [file pharmaceuticals-15-01582-s001.zip › pharmaceuticals-2049419-supplementary.pdf]

Supplementary metarials

Kaempferol interferes with varicella-zoster virus replication in human foreskin fibroblasts

Contents

Figure S1. Effects of acyclovir on VZV replication
Figure S2. Effects of kaempferol on VZV lytic gene expression

Figure S1. Effects of acyclovir on VZV replication

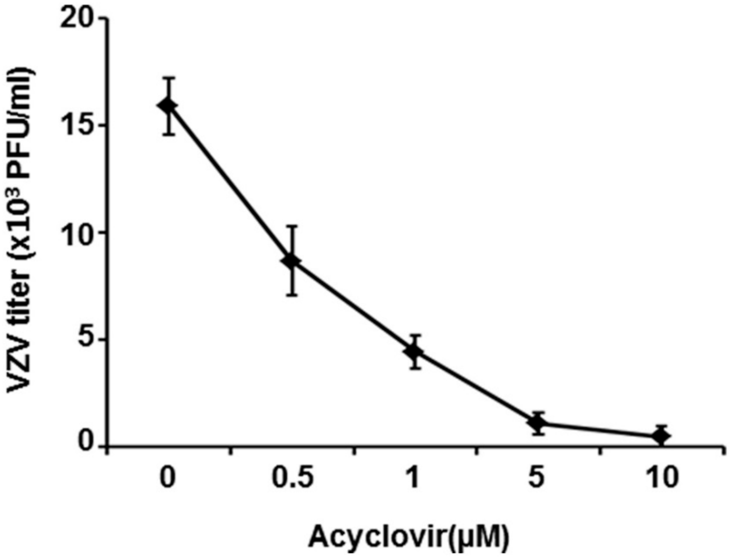

HFFs were inoculated with serially diluted cell-associated VZV-YC01 and treated with acyclovir at the indicated concentrations. Cells were re-treated with the same concentrations of acyclovir 3 d after inoculation. At 6 d after inoculation, cells were stained with 0.3% crystal violet and the number of plaques was counted.

Figure S2. Effects of kaempferol on VZV lytic gene expression.

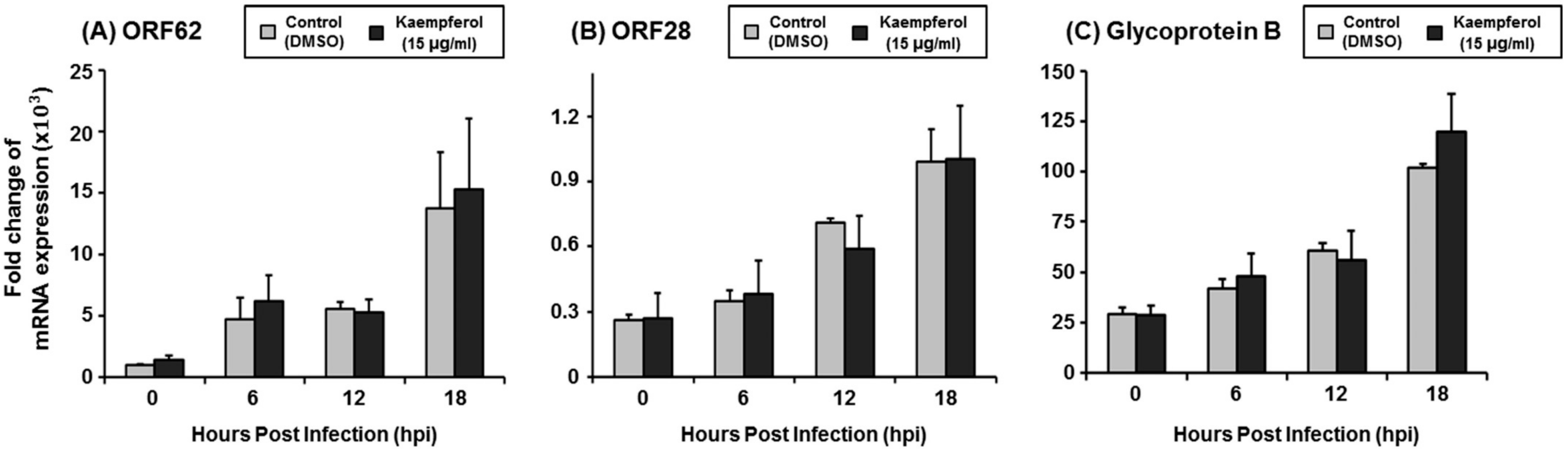

HFFs were inoculated with cell-associated VZV-YC01 at a MOI of 0.1 and treated with DMSO or kaempferol (15 μg/ml). At 0, 6, 12, and 18 h after inoculation, total RNA was extracted and reverse-transcribed into cDNA. The mRNA levels of (A) ORF62 (IE), (B) ORF28 (E) and (C) Glycoprotein B (L) were measured via qRT-PCR. Experiments were performed in triplicate, and values were expressed relative to those in uninfected cells (defined as 1).
